# Tuning down the hedonic brain: Cognitive load reduces neural responses to high-calorie food pictures in the nucleus accumbens

**DOI:** 10.3758/s13415-018-0579-3

**Published:** 2018-03-14

**Authors:** Lotte F. van Dillen, Henk van Steenbergen

**Affiliations:** 1 Institute of Psychology, Leiden University, P.O. Box 9555, 2300 RB Leiden, The Netherlands; 2 Leiden Institute for Brain and Cognition, Leiden University, Leiden, The Netherlands

**Keywords:** Reward, Nucleus accumbens, Cognitive load, Food

## Abstract

The present research examined whether cognitive load modulates the neural processing of appetitive, high-calorie food stimuli. In a functional magnetic resonance imaging (fMRI) study, participants quickly categorized high-calorie and low-calorie food pictures versus object pictures as edible or inedible while they concurrently performed a digit-span task that varied between low and high cognitive load (memorizing six digits vs. one digit). In line with predictions, the digit-span task engaged the dorsolateral prefrontal cortex (DLPFC) when cognitive load was high compared to low. Moreover, exposure to high-calorie compared to low-calorie food pictures led to increased activation in the nucleus accumbens (NAcc), but only when cognitive load was low and not when it was high. In addition, connectivity analyses showed that load altered the functional coupling between NAcc and right DLPFC during presentation of the high-calorie versus low-calorie food pictures. Together, these findings indicate that loading the cognitive system moderates hedonic brain responses to high-calorie food pictures via interactions between NAcc and DLPFC. Our findings are consistent with the putative cognitive nature of food motivation. Implications for future research are discussed.

**Electronic supplementary material:**

The online version of this article (10.3758/s13415-018-0579-3) contains supplementary material, which is available to authorized users.

The intricate relationship between emotion and cognition is a topic of continuing debate. For example, in the context of food research opinions differ as to whether hedonic responses to appetitive cues have a reflexive or a cognitive basis (Heatherton & Wagner, 2011; Hofmann & Van Dillen, 2012; Kavanagh, Andrade, & May, 2005). The present study builds on the idea that hedonic responses are, at least to some extent, shaped by higher-order cognitive functions (Hofmann & Van Dillen, 2012; Kavanagh et al., 2005). In earlier work, we have proposed a working memory account of affective processing, which posits that affective and cognitive processes rely on the same limited mental resources (Van Dillen & Koole, 2007). Accordingly, whenever a cognitive task requires more mental resources, fewer resources are left for affective processing. In line with this view, behavioral studies have shown that loading people’s mental resources with a cognitive task reduces selective attention to attractive food options and opposite-sex faces (Van Dillen, Papies, & Hofmann, 2013), as well as (naturally occurring) food cravings (Kemps, Tiggemann, & Grigg, 2008; Skorka-Brown, Andrade, & May, 2014), alcohol-related approach tendencies (Sharbanee, Stritzke, Jamalludin, & Wiers, 2014), and craving-induced consumption choices (Van Dillen & Andrade, 2016; Van Dillen et al., 2013).

In the present study, we used functional magnetic resonance imaging (fMRI) to investigate the neural basis of processing food pictures under different levels of cognitive load. Previous neuroimaging findings using affective stimuli unrelated to food have suggested that loading mental resources with a concurrent task reduces neural activity in limbic regions in response to aversive stimuli (Kanske, Heissler, Schönfelder, Bongers, & Wessa, 2011; Pessoa, McKenna, Gutierrez, & Ungerleider, 2002; Van Dillen, Heslenfeld, & Koole, 2009) as well as reward areas to positively valenced scenes (Erk, Kleczar, & Walter, 2007; Kanske et al., 2011). In the present study, we used fMRI to provide evidence for a similar modulation by examining neural responses to high-calorie compared with low-calorie food pictures when presented in the context of different cognitive loads. Extending previous findings to the domain of food addresses the question of whether the processing of appetitive (food) stimuli is similarly resource dependent or whether this is more reflexive in nature and contributes to a better understanding of the nature of hedonic consumption and how it can be regulated.

Previous work has shown that food pictures reliably engage reward-processing areas of the brain, most notably the nucleus accumbens (NAcc; Berridge, 2009; for a meta-analysis, see Van der Laan, De Ridder, Viergever, & Smeets, 2011), and that high-calorie food pictures trigger a stronger response in the NAcc than low-calorie food pictures do (Goldstone et al., 2009). EEG findings further suggest that prefrontal cortical areas play an important role during later stages of food-related differentiations (starting at around 300 ms; Toepel, Knebel, Hudry, le Coutre, & Murray, 2009), and it has been shown that specific categorization instructions can modify blood-oxygen-level-dependent (BOLD) responses to images of high-calorie foods in reward-processing areas (Siep et al., 2009).

Building on this research, we expected that responses to food rewards in the NAcc will partly rely on, and interact with, higher-order cognitive processes involved in the prefrontal cortex. In order to test this prediction, participants in the present experiment were asked to categorize pictures of high-calorie (i.e., high reward) and low-calorie (i.e., low reward) food items, as well as nonfood objects, as edible or inedible in a speeded manner (Toepel et al., 2009; Van Dillen et al., 2013). At around the same time, cognitive load was manipulated on a trial-by-trial basis by means of a digit-span task (Sternberg, 1966), which was expected to engage the working-memory network, including the dorsolateral prefrontal cortex (DLPFC; D’Esposito & Postle, 2015; Palva, Monto, Kulashekhar, & Palva, 2010; Rypma, Prabhakaran, Desmond, Glover, & Gabrieli, 1999). In line with previous findings, we expected greater reward processing of high-calorie food pictures compared with low-calorie food pictures, as reflected by greater responses to these pictures in the NAcc (Demos, Heatherton, & Kelley, 2012; Van der Laan et al., 2011). Critically, this responsivity was expected to be modulated by concurrent cognitive load, such that, in comparison to low load, high load should reduce the NAcc activity to high-calorie versus low-calorie foods. We also used psychophysiological interaction (PPI) analyses to examine the effect of cognitive load on functional connectivity between the NAcc and the dorsolateral PFC.

## Method

### Participants and design

The data of 29 volunteers at Leiden University (13 males and 16 females, *M*_Age_ = 21.03 years, *SD* = 3.02 years) were analyzed. The data of an additional five participants had to be discarded, due to extreme movement (*N* = 4) and a failure in data storage (*N* = 1). All 29 participants were right-handed and native Dutch speakers who did not report any history of neurological or psychiatric problems. In addition, we verified that they were not currently on a diet, had a normal body mass index (BMI; computed for each participant by dividing their weight in kilograms by the square of their length in meters), and had eaten between 3 hours and at maximum 1 hour prior to the experiment, as research suggests that all of these factors can influence neural processing of food rewards (Burger & Stice, 2011; Frank et al., 2010; Siep et al., 2009; Stice, Burger, & Yokum, 2013; Stoeckel et al., 2008). Participants provided written informed consent (according to the Declaration of Helsinki) after the study procedure had been explained to them and were paid €25 for participation at the end of the study. The study was approved by the Medical Ethics Committee of Leiden University.

The experimental design was a 2 (cognitive load: high vs. low) × 3 (picture type: high-calorie food, low-calorie food, nonfood objects) factorial design, both factors within participants. Dependent measures were participants’ performance on the digit-span task and food-categorization task (accuracy scores and reaction times), and brain activity (see below) time-locked to the digit-span task and the food pictures during the food-categorization task. How we determined all data exclusions, all manipulations, and all measures in the study description are provided below.

### Procedure and equipment

Participants were invited to the lab to participate in a brain-imaging experiment. Before starting with the actual task, they were instructed about the experimental setup and answered a series of control questions. They indicated how hungry they were at that moment on a 9-point scale, ranging from 1 (*not at all*) to 9 (*very much*), and how long ago they had eaten (in hours). In addition, participants filled out the Power of Food Scale (PFS; Lowe et al., 2009), a validated measure of psychological sensitivity to food rewards. The scale contains 15 items (such as “I find myself thinking about food even when I’m not physically hungry” and “If I see or smell a food I like, I get a powerful urge to have some”) and involves responses on a Likert scale ranging from 1 (*don’t agree at all*) to 5 (*strongly agree*).

**Fig. 1:**
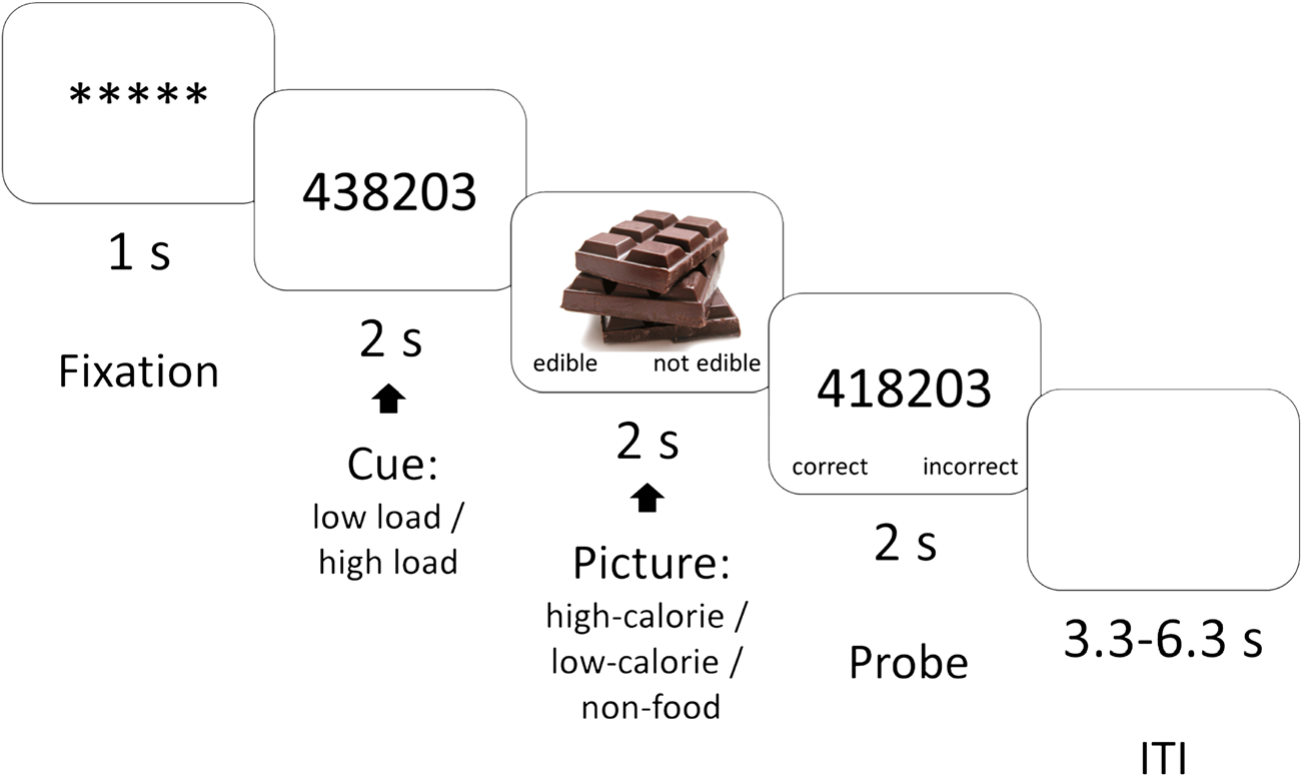
Example of a trial from the task that combined a digit-span manipulation with a food-categorization task. Analyses focused on brain activity during cue onset (effect of cognitive load) and during picture presentation (effect of cognitive load on processing high-calorie vs. low-calorie food pictures). ITI = intertrial interval

Participants were then led to the scanner room and positioned supine in the MRI scanner, where they completed the actual experiment. All stimuli were back-projected onto a screen and viewed by participants through an angled mirror. As illustrated in Fig. 1, the experiment consisted of a picture-categorization task in which participants quickly decided whether the displayed image was edible or inedible (Toepel et al., 2009). We selected two sets of food pictures: one set that contained 25 pictures of high-calorie foods and one set that contained 25 pictures of low-calorie foods. Previous research (Van Dillen et al., 2013) has demonstrated that compared to the pictures of the low-calorie foods, the pictures of the high-calorie foods are perceived as more attractive, and arouse stronger cravings. A set of 25 pictures of food-unrelated, inedible objects (e.g., a telephone, a vase; Van Dillen et al., 2013) was used as filler trials for the categorization task and to prevent quick habituation to the food pictures.

The experiment contained two blocks of 75 experimental trials each (150 trials in total) with a self-paced break in between blocks. Within blocks, half of the images of each picture subset (high/low calorie foods and nonfood objects) were presented under high concurrent load, and half were presented under low concurrent load. When images were presented under high load in the first block, they were presented under low load in the second block, and vice versa. Within blocks, the six trial types (i.e., high/low cognitive load paired with a picture of a high/low-calorie food or nonfood object) were presented in random order. The order of the blocks was moreover counterbalanced between participants. All 75 pictures were thus presented to participants twice: once under high cognitive load and once under low cognitive load, but the order in which specific images were paired with high versus low cognitive load varied between participants.

We varied cognitive load by means of a digit-span manipulation (Sternberg, 1966) such that while performing the categorization task, participants were instructed to rehearse either a one-digit number (low load), or a seven-digit number (high load). As shown in Fig. 1, following the row of asterisks that announced the beginning of a trial (duration one second), participants first viewed for 2 seconds the number that they had to retain during the remainder of the trial. Then, the food picture appeared on-screen and participants had 2 seconds to categorize the picture as edible or inedible. This way, we ensured that participants would focus their attention on the content of the picture and would not engage in avoidant gaze strategies in order to reduce interference with the digit-span task (Siep et al., 2009; Van Dillen & Derks, 2012; Van Reekum et al., 2007). When participants had provided their response to the picture, a number was again presented on-screen, and participants had 2 seconds to judge whether it was the same number as they had retained. In half of the trials, this was the same number participants had seen previously, whereas in the other half, one of the digits was different. In the high-load trials, we varied the position of the digit that differed, except for the digits at both ends, such that participants had to retain the full sequence in order to arrive at the correct answer (Van Dillen & Derks, 2012). In the low-load trials, only one number was presented. For half of the participants, the right button represented the correct response and the left button the incorrect response, while for the other participants this order was reversed.

To avoid systematic overlap of BOLD responses within and between trials, the interval between the response window of the Sternberg task and the beginning of the next trial was jittered using a random duration that varied between 3,300 and 6,300 ms in steps of 200 ms. The intertrial interval accordingly varied between 10.3 and 13.3 seconds.

Prior to the presentation of the first block, participants were given a block of 16 practice trials to get familiar with the experimental set-up and the scanner. After the scan session, participants were taken out of the scanner and guided to an adjacent interview room where they were again presented with the pictures of the food items used in the categorization task and were asked to rate on a Likert scale, ranging from 1 (*not at all*) to 9 (*very much*), how tasty they thought each of the pictures looked. Participants were then thanked for their efforts, paid, and debriefed.

A personal computer controlled presentation of the experimental trials and recorded participants’ responses. The experimental trials were presented in E-Prime 2.0 (Psychology Software Tools, Inc., Pittsburgh, PA, USA). Participants responded by using a scanner-compatible button box attached to their upper legs.

### MRI data acquisition

Scanning was performed with a standard whole-head coil on a 3-T Philips Achieva MRI system (Best, The Netherlands) in the Leiden University Medical Center. During the task, two runs of at least 420 T2*-weighted whole-brain EPIs were acquired, including two dummy scans preceding the scan to allow for equilibration of T1 saturation effects (TR = 2.2 s; TE = 30 ms, flip angle = 80°, 38 transverse slices, 2.75 × 2.75 × 2.75 mm +10% interslice gap). Stimuli were projected onto a screen that was viewed through a mirror at the head end of the scanner. After the functional runs, a high-resolution EPI scan (flip angle = 80°, 84 transverse slices, 1.964 × 1.964 × 2 mm) and a B0 field map were acquired for registration purposes. This was followed by a 3-D T1-weighted scan (TR = 9.8 ms; TE = 4.6 ms, flip angle = 8°, 140 slices, 1.166 × 1.166 × 1.2 mm, FOV = 224.000 × 177.333 × 168.000).

### Behavioral analyses

Descriptives (means and standard errors) of accuracy rates and reaction times as a function of block (1, 2), cognitive load (high, low) and picture type (high-calorie vs. low-calorie food) can be found in Table 1. The analyses scripts and raw data can be found on the OSF link (https://osf.io/e9pjm).

**Table 1:** Performance on the digit-span task and food-categorization task

			Digit span		Food categorization	
			RTs	Accuracy	RTs	Accuracy
High load	High cal.	Block 1	1,186 (27)	69 (2)	717 (22)	95 (3)
		Block 2	1,155 (28)	59 (3)	638 (21)	98 (1)
	Low cal.	Block 1	1,185 (28)	65 (2)	763 (21)	94 (4)
		Block 2	1,128 (27)	67 (3)	671 (20)	97 (2)
Low load	High cal.	Block 1	770 (27)	98 (1)	745 (21)	95 (3)
		Block 2	695 (28)	82 (7)	656 (22)	98 (1)
	Low cal.	Block 1	790 (28)	96 (1)	775 (21)	94 (3)
		Block 2	687 (27)	83 (6)	674 (21)	98 (1)

Means and standard errors (between brackets) for reaction times (RTs) in milliseconds and accuracy scores in percentages, and as a function of cognitive load (high, low), target picture (high-calorie vs. low-calorie food). and block (first, second)

We analyzed participants’ performance on the working-memory task and the food-categorization task, as well as their attractiveness ratings of the food pictures. For all analyses we used a significance threshold of *p* < .05. Nonfood trials containing object pictures were included for the categorization task and to prevent quick habituation to the food pictures but were not of focal interest. Moreover, because nonfood trials differed from the food trials both in content and in frequency, it is difficult to interpret behavioral and neural differences between these trial types. For reasons of completeness, analyses including nonfood trials are reported in the Supplementary Materials. Note that the inclusion of nonfood trials did not significantly alter the pattern of behavioral findings described in the following.

To analyze accuracy data within participants for both tasks, we conducted a generalized estimated equation analysis (GEE; IBM SPSS Statistics Version 23), which can be used for categorical dependent variables with a binomial probability distribution and a logit link function. Accuracy scores were analyzed with food type, load, and block as fixed factors, and subject as a random factor.

Reaction times for both tasks were analyzed using the linear mixed-effects models procedure run in SPSS (IBM SPSS Statistics Version 23) and an autoregressive covariance structure. Mixed-effects models account for correlations between repeated measures with greater flexibility than more traditional repeated-measures analyses, as they include both fixed and random effects and allow for different target distributions and the inclusion of all data points instead of aggregations across multiple trials. Participants’ reaction times on accurate trials were analyzed with all combinations of stimulus type, load, and block as fixed factors as well as random slopes in addition to the random intercept for subjects.

Finally, we analyzed the tastiness ratings of the food pictures that were provided at the end of the experiment to test whether high- calorie food items were perceived as tastier compared to low-calorie food items using a linear mixed-effects model, with food type as the predictor and a random intercept for subjects and an autoregressive covariance structure.

All the above analyses were additionally repeated with the standardized scores of BMI, PFS, or self-reported hunger as a covariate added to the model.

### FMRI preprocessing

FMRI data analysis was carried out using FEAT (FMRI Expert Analysis Tool) Version 6.00, part of FSL (FMRIB’s Software Library; www.fmrib.ox.ac. uk/fsl; Smith et al., 2004). For each scan, we cut the volumes at the end of the scan in which no task-related brain activation was acquired, resulting in 420 volumes used for all runs and participants. Data were first preprocessed, which involved motion correction, spatial smoothing using a Gaussian kernel of FWHM 6.0 mm, grand-mean intensity normalization of the entire 4-D data set by a single multiplicative factor, and registration. Scans were first registered to high-resolution EPI images, which were registered to T1 images, which in turn were registered to the standard space of the MNI (Montreal Neurological Institute) with 2 mm resolution using FNIRT (warp resolution 10 mm). This preprocessed data was then used by ICA-AROMA (ICA-based Automatic Removal of Motion Artifacts) to remove motion-related artefacts (Pruim, Mennes, van Rooij, et al., 2015). This recently developed method minimizes the impact of motion similarly as scrubbing and spike regression and limits the loss in temporal degrees of freedom, thus increasing statistical power for the analyses (Pruim, Mennes, Buitelaar, & Beckmann, 2015). The denoised functional data was then submitted to FEAT to run brain extraction, high-pass temporal filtering (Gaussian-weighted least-squares straight line fitting, with sigma = 50.0 s) and registration. In native space, the fMRI time series were analyzed using an event-related approach in the context of the general linear model with local autocorrelation correction. All models were also high-pass-filtered (Gaussian-weighted least-squares straight-line fitting, with sigma = 50.0 s).

Our event-related model included two regressors time-locked to the cue presentations (high load, low load) and six regressors time-locked to the picture presentations (high calorie, low calorie, nonfood × two levels of load; see Fig. 1 for an overview of the trials in the task). The probe stimulus was modelled as a nuisance regressor. Brain activity related to cognitive load was assessed using the contrast high-load cue minus low-load cue. Brain activity related to a modulation of hedonic processing by cognitive load was assessed using the interaction contrast [high-calorie picture > low-calorie picture]_low load_ > [high-calorie picture > low-calorie picture]_high load_. In addition, we assessed the interaction contrast [high-calorie picture > nonfood picture]_low load_ > [high-calorie picture > nonfood picture]_high load_. Finally, we assessed the effect of load on food pictures (combining low-calorie and high-calorie pictures) versus nonfood pictures, using the contrast [food picture > nonfood picture]_low load_ > [food picture > nonfood picture]_high load_. Analyses on main effects of picture type independent of load are described in the Supplementary Materials.

Note that even though there is no jitter between the cue and picture, the interaction contrasts mentioned above are statistically independent of the main effect of load associated with the cue preceding the picture. At the same time, however, main effects of load *during picture presentation* are confounded by temporal autocorrelation between the cue and picture events. This implies that it is not valid to compare brain activity to the pictures as far as this concerns main effects of load (i.e., comparisons between the two load conditions). For this reason, in the extracted brain data from our interaction contrast (see Fig. 3 and Fig. S2) we only report and interpret comparisons (simple effects) *within* the two load conditions.

We also built a functional connectivity model to test for psychophysiological interaction (PPI) between the NAcc (physiological variable) and the dorsolateral prefrontal cortex (DLPFC) when different food pictures were presented under high versus low load (psychological variable). The physiological regressor for this PPI model used extracted time-course information based on a sphere (radius 6 mm) that included the peak of left NAcc activation (*x* = −12, *y* = 14, *z* = −8), that was identified by the analysis of cognitive load on hedonic processing using the earlier described interaction contrast (see Results). The convolved psychological regressor represented the following contrasts: [high-calorie picture > low-calorie picture]_high load_ > [high-calorie picture > low-calorie picture]_low load_. The PPI regressor was computed as the product of the demeaned physiological time course and the centered psychological regressor (O’Reilly, Woolrich, Behrens, Smith, & Johansen-Berg, 2012). Following standard recommendations, a separate main effect regressor of the psychological variable was added in order to partition out shared variance. Nuisance regressors for the remaining events were also modeled. Please note that because the psychological regression was added to our model, our PPI contrast reflects altered functional connectivity over and above alterations in mean activation (O’Reilly et al., 2012).

For all models, the trial-type regressors used square-wave functions time-locked to the onset and offset of the respective stimulus which were convolved with a canonical HRF and its temporal derivative. After confirming that individual runs were registered correctly and did not indicate excessive motion, the relevant contrasts were combined across runs on a subject-by-subject basis using fixed-effects analyses. Second-level contrast images in standard space were merged into a single 4-D file for nonparametric voxelwise permutation-based statistical testing using FSL randomise (see below).

### MRI statistical analyses and thresholding

In order to test our hypotheses concerning the effect of cognitive load on hedonic processing of high-calorie versus low-calorie food pictures, the main analyses described in this study focused on small, anatomically defined volumes of interest. We used an anatomical mask of bilateral DLPFC (“middle frontal gyrus” in Automated Anatomical Labeling Atlas; for a similar approach, see Li, Chen, Han, Chui, & Wu, 2012, for the analyses concerning cognitive load. We used an anatomical mask of bilateral NAcc (“Accumbens” in the Harvard-Oxford Subcortical Structural Atlas) for the analysis of load on hedonic processing. The PPI analysis aimed to test for altered functional connectivity between NAcc and working-memory-related brain activity. To maximize statistical power to detect effects, this analysis was limited to a functionally defined volume of interest based on the cluster of brain activity that was most strongly engaged by high relative to low cognitive load (see Results). Note that the choice of this region is independent of the PPI analyses (Kriegeskorte, Simmons, Bellgowan, & Baker, 2009), because the latter reveals functional connectivity effects over and above mean activation.

We report results within these masks that are corrected for multiple comparisons using FSL randomise (Winkler, Ridgway, Webster, Smith, & Nichols, 2014), a nonparametric method that uses the observed null distribution of the max cluster size (based on 5,000 permutations). Statistical maps were based on a height threshold of T > 2.3 and a cluster-corrected probability of P < 0.05, unless otherwise noted. Note that the use of relatively low cluster-forming threshold in parametric analyses normally inflates the rate of false positives. However, this limitation does not apply to the nonparametric method used here (Eklund, Nichols, & Knutsson, 2016).

For reasons of completeness, we also reported whole-brain analyses for all contrasts described. Because the whole-brain analyses for the cognitive load effect produced clusters spanning multiple anatomical regions, this analysis was limited to gray matter voxels and following earlier recommendations (Woo, Krishnan, & Wager, 2014) using a more stringent height threshold of T > 4.1, again combined with a cluster-corrected probability of P < 0.05. All brain images in the figures show cluster-corrected brain activity overlaid on an MNI standard brain with 2 mm resolution, displayed according to radiological convention (left part of image is right part of brain). Unthresholded statistical maps of all contrasts are available on NeuroVault (Gorgolewski et al., 2015; https://neurovault.org/collections/3285/).

## Results

### Behavioral results

#### Individual differences

Participants’ average BMI was *M* = 22.32, *SD* = 1.85, range 19.39–26.88, with one participant scoring above the normal range of 18–25. Mean self-reported hunger was *M* = 3.28, *SD* = 1.51, mean last time eaten was *M* = 1.13 hours, *SD* = .41 hours. The mean power of food sum score was *M* = 57, *SD* = 13, range 28–87, and the scale had good reliability, with Cronbach’s alpha = .89.

#### Digit-span task

Table 1 depicts means and standard errors for reaction times and accuracy scores on the digit span task and food categorization task.

The GEE analysis of the accuracy scores on the digit span task revealed significant main effects of load, Wald (1) = 56.62, *p* < .001, and block, Wald (1) = 9.10, *p* = .003. Participants on average successfully retrieved 93% of the one-digit numbers (*SE* = 1.9%) and 65% of the seven-digit numbers (*SE* = 2%), confirming the effectiveness of our cognitive load manipulation. Participants moreover made more accurate responses in the first block (*M* = 89%, *SE* = 1.9%) compared to the second block (*M* = 74%, *SE* = 5.6%) indicating that digit-span performance deteriorated significantly over time. There was also an interaction effect of load and block, Wald (1) = 9.28, *p* = .002, such that over time, accuracy decreased more on the low-load trials (*d* = −15%, *SE* = 7.2%, *p* = .036, CI [−29, −1] than on the high-load trials (*d* = −3%, *SE* = 2.7%, *p* = .324, CI [−8, 3]. There was moreover an interaction effect of food type and block, Wald (1) = 8.97, *p* = .003, such that over time, accuracy decreased more on trials with high-calorie food pictures (*d* = −19%, *SE* = 6.1%, *p* = .001, CI [−31, −1] than on trials with low-calorie food pictures (*d* = −11%, *SE* = 5.9%, *p* = .063, CI [–23, 1].

Mixed-model analyses of the reaction times on the digit-span task revealed main effects of load, *F*(1, 80.56) = 585,96, *p* < .001, and block, *F*(1, 80.56) = 15,57, *p* < .001. Participants were slower to respond to the seven-digit series (*M* = 1,163 ms, *SE* = 22 ms) than to the one digit (*M* = 735 ms, *SE* = 22 ms), and became faster from the first block (*M* = 982 ms, *SE* = 22 ms) to the second block (*M* = 916 ms, *SE* = 22 ms).

#### Food-categorization task

The GEE analysis of participants’ food categorization accuracy scores revealed no significant effects, suggesting that performance (*M* = 95%, *SE* = .32%) was stable across load, target category, and time.

A linear mixed-model analysis of participants’ reaction times on the food categorization task revealed significant main effects of food type, *F*(1, 81.24) = 11.31, *p* = .001, and block, *F*(1, 81.24) = 94.15, *p* < .001. Participants were faster to categorize high-calorie food pictures (*M* = 686 ms, *SE* = 22 ms) than low-calorie food pictures (*M* = 720 ms, *SE* = 21 ms, CI [−53, −14]), and made faster categorizations in the second block (*M* = 655 ms, *SE* = 22 ms) compared with the first block (*M* = 752 ms, *SE* = 22 ms, CI [−115, −77]).

When power of food scores, self-reported hunger, or BMI were included as covariates in the above analyses, this yielded no additional significant effects and did not alter the above-reported pattern of findings.

#### Tastiness ratings of the food pictures

Two participants failed to provide the tastiness ratings assessed after the scan session, leaving 27 participants for this analysis. Tastiness ratings ranged from 1 to 9, thus covering the full response scale. There was a significant effect of food type, *F*(1, 2375) = 249.87, *p* < .001), confirming that participants perceived the high-calorie food pictures as tastier (*M* = 6.66, *SE* = .30) than the low-calorie food pictures (*M* = 5.39, *SE* = .13; CI [1.10, 1.42]).

When we added standardized power of food scores as covariate to the model, we moreover observed a significant interaction between food type and power of food, *F*(1, 2274.97) = 70.78 , *p* < .001. There was a significant positive relationship between power of food scores and the ratings of high-calorie foods, *B* = .54, *SE* = .14, *t*(29.72) = 3.83, *p* = .001, CI [.25, .82], but not low-calorie foods, *B* = −.20, *SE* = .14, *t*(29.72) = –1.40, *p* = .171, CI: [−.48, .90].

Including self-reported hunger or BMI as covariates yielded no additional significant effects and did not change the above-reported pattern of findings.

### Functional MRI results

#### Cognitive load involves DLPFC

**Fig. 2:**
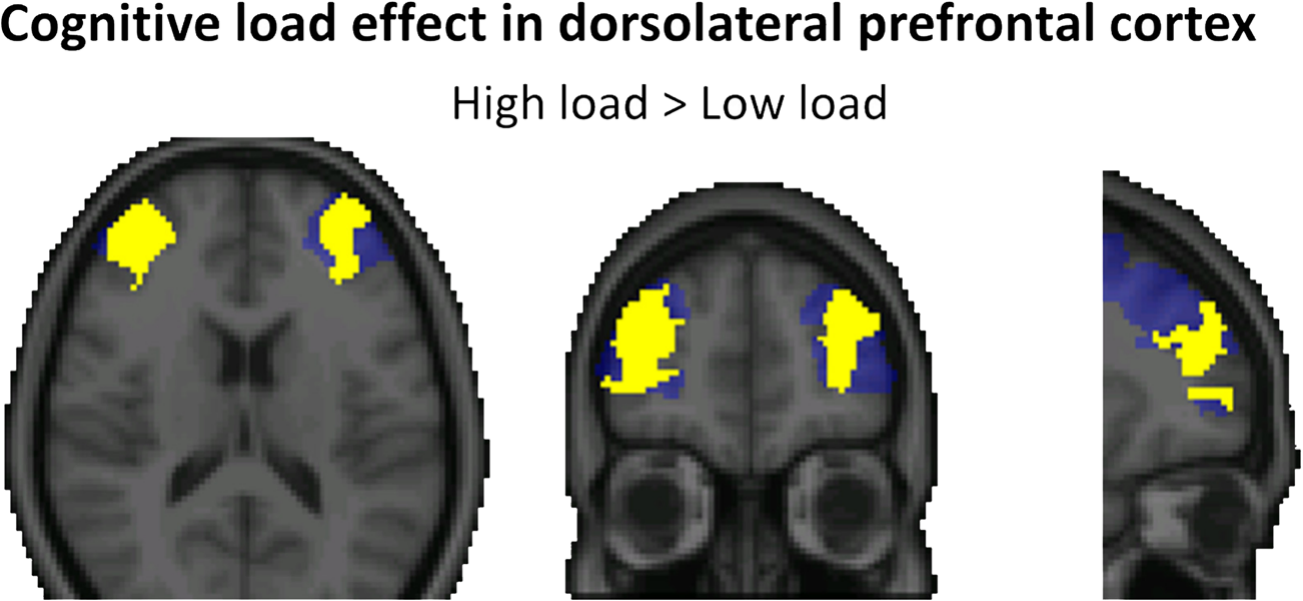
High versus low cognitive load increased brain activity (slices at *x* = 30, *y* = 50, *z* = 16 mm) in response to the digit span cue in bilateral DLPFC. Maps were cluster corrected within an anatomical mask of bilateral DLPFC (depicted in blue), *p* < .05. Results of the whole-brain analyses for this contrast are presented in Table 2. (Color figure online)

We first probed for brain activity that increased during the high versus low load in response to the working memory cue in the DLPFC (see Fig. 2). As predicted, we observed an effect of cognitive load, that was the strongest in the right DLPFC (peak: *x* = 34, *y* = 60, *z* = −2 mm; *p* < 0.001; extent = 2,090 voxels). Two additional clusters of brain activity were found in left DLPFC (peak: *x* = −34, *y* = 42, *z* = −2 mm; *p* = 0.002; extent = 1,150 voxels) and a more posterior part of right DLPFC (peak: *x* = 34, *y* = 0, *z* = 36 mm; *p* = 0.011; extent = 650 voxels). For reasons of completeness, Table 2 reports the results of an additional whole-brain analysis for this contrast. This analysis confirmed that cognitive load engaged the entire working memory network, including cingulate, parietal, and occipital cortices (Duncan & Owen, 2000; Kanske et al., 2011; Rypma et al., 1999; Van Dillen et al., 2009).

**Table 2:** Whole-brain analysis on high > low cognitive load

Areas	Cluster size (voxels)	*p* value of max	Peak MNI coordinates		
			*x*	*y*	*z*
Left pre/postcentral gyrus, precuneus occipital cortex, cingulate cortex	13,684	<.001	−30	−42	−28
Cingulate gyrus	1,306	<.001	6	30	20
Right frontal pole, middle and inferior frontal gyrus	322	.001	36	48	8
Left putamen	222	.001	−28	−10	−12
Right thalamus	164	.001	10	−10	4
Superior and middle temporal gyrus, supramarginal gyrus	134	.002	56	−22	−12
Left thalamus	123	.002	−6	−22	4
Left caudate	107	.002	−12	12	2
Frontal pole	90	.003	−32	52	14
Cerebellum	65	.007	16	−50	−24
Posterior cingulate gyrus	51	.008	−14	−20	38
Right postcentral gyrus	45	.009	66	−12	20
Right planum temporale	38	.013	44	−30	10
Right temporal gyrus	27	.023	48	−36	−2
Left frontal pole	27	.023	−26	46	−14
Left parietal operculum cortex	24	.029	−42	−32	16
Right thalamus	24	.029	22	−30	−6
Right temporal pole	22	.034	54	12	−14
Left planum temporale	17	.05	−42	−30	6
Right frontal pole	17	.05	26	58	−14

Table shows clusters with height threshold of T > 4.1 and a cluster-corrected probability of *p* < .05

#### Modulation of reward processing by load in NAcc

**Fig. 3:**
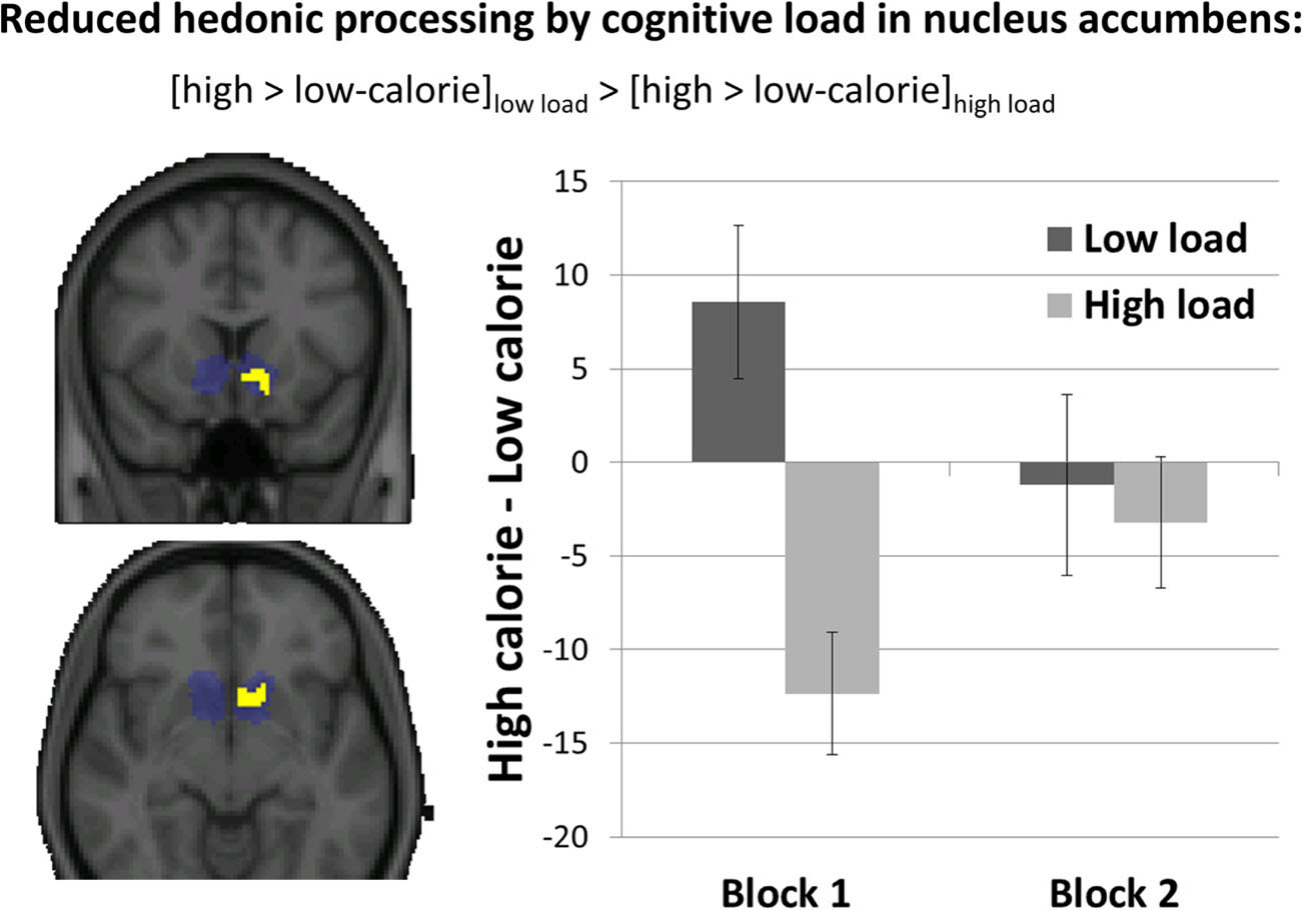
Reduction of brain activity to high-calorie versus low-calorie food pictures by high versus low cognitive load in left NAcc (slices at *y* = 14, *z* = −8 mm), cluster corrected using anatomical mask of bilateral NAcc (depicted in blue), *p* < .05. Bar graph shows the extracted differences in brain activity (in arbitrary units) from this cluster. Error bars represent standard errors of the difference scores. (Color figure online)

We next investigated whether cognitive load reduced the impact of high versus low calorie food in the NAcc, a region that plays a central role in hedonic processing. Confirming our hypothesis, as Fig. 3 shows, cognitive load was observed to reduce brain activity to the high-calorie versus low-calorie food pictures in the left NAcc (peak: *x* = −14, *y* = 14, *z* = −14 mm; *p* = 0.035; extent = 64 voxels). Subsequent examinations of the extracted brain activity through a repeated-measures analyses of variance additionally yielded a significant three-way interaction between food type, load, and block, *F*(1, 27) = 4.57, *p* = .042. Comparisons of the estimated marginal means revealed that the modulatory effect of load on NAcc responses to high-calorie compared to low-calorie food pictures was driven by an effect in the first block, *F*(1, 27) = 22.03, *p* < .001, but was absent in the second block, *F*(1, 27) < 1, *p* > .770. Consistent with the predicted modulation of reward processing, NAcc response in the first block was stronger for high-calorie than for low-calorie pictures under low load. Interestingly, this effect reversed under high load. Including Power of Food, self-reported hunger, or BMI as covariates yielded no additional significant effects and did not change the above-reported pattern of findings. For reasons of completeness, the Supplementary Materials reports an analysis on the extracted brain activity for the NAcc cluster as a function of all levels of food type (including the nonfood pictures), load, and block.

The interaction contrast comparing the effect of load on high-calorie versus nonfood pictures did not yield significant clusters. Neither did the interaction contrast comparing the effect of load on food versus nonfood pictures. Exploratory whole-brain analyses for the contrasts reported above and their inversions did not reveal modulation in other brain regions

#### Functional interactions between NAcc and working-memory-related DLPFC during food pictures

**Fig. 4:**
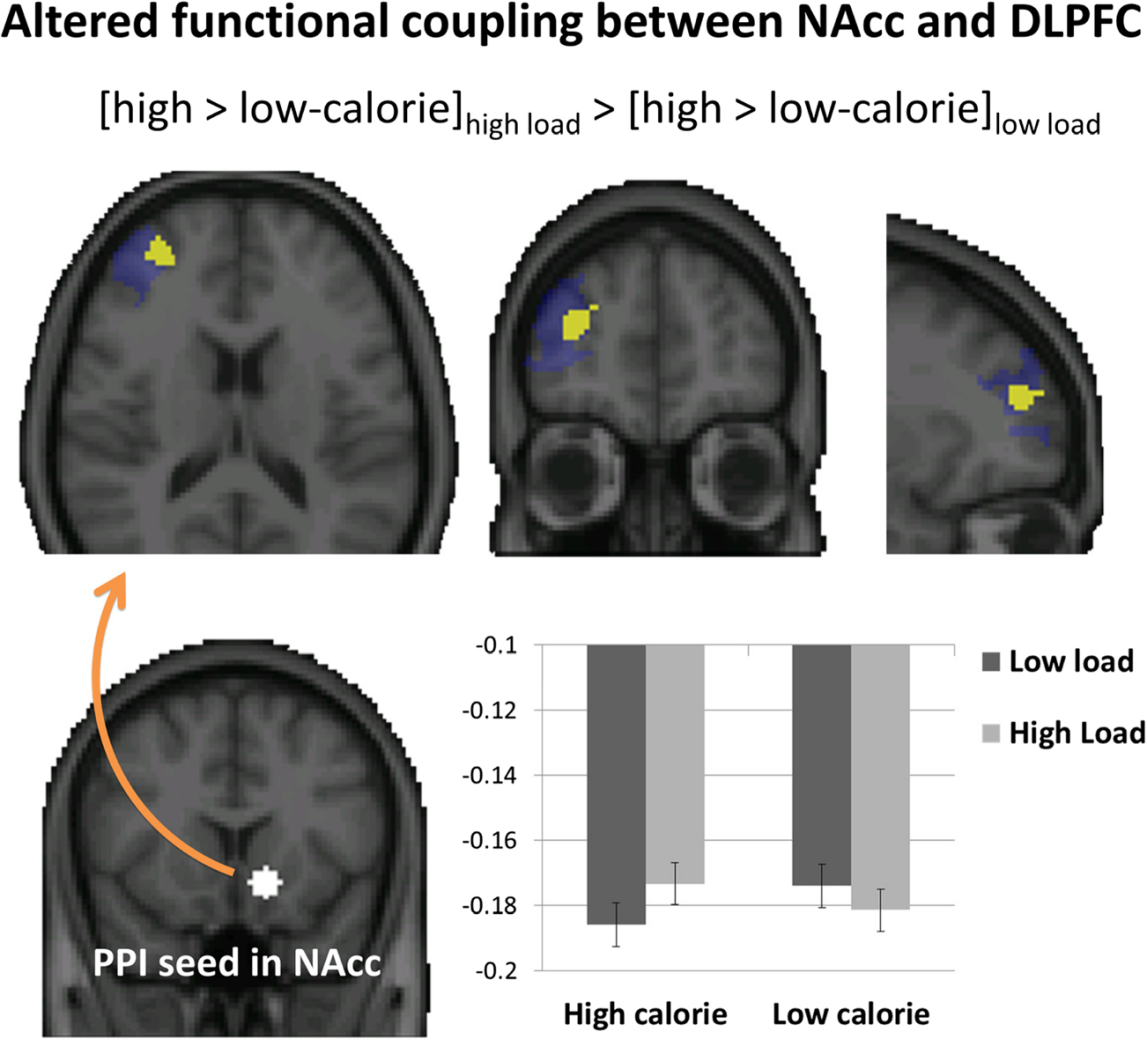
Altered functional connectivity between NAcc (slice at *y* = 14) and the right DLPFC (slices at *x* = 30, *y* = 50, *z* = 16 mm) during the presentation of the high-calorie versus low-calorie food pictures in the context of high versus low cognitive load. Contrast shows functional connectivity values cluster corrected using a functional mask of cognitive load effect in right DLPFC depicted in blue (see Results), *p* < .05. Bar graph shows extracted functional connectivity values (in arbitrary units.) from this cluster. Error bars represent standard errors of the difference scores calculated within the two load conditions. (Color figure online)

Finally, we tested for differences in functional connectivity between the NAcc and the DLPFC when participants processed the food pictures using a PPI analysis. We expected that cognitive load should change the functional coupling between these regions specifically during the processing of high compared to low-calorie food pictures. To test this hypothesis, we ran a PPI analysis that tested for increased functional connectivity between the NAcc (sphere of 6 mm centered on *x* = −12, *y* = 14, *z* = −8 mm) and the activity in the cluster in right DLPFC that was shown to be most strongly engaged by the cognitive load manipulation. We chose this small region in order to maximize statistical power to detect effects. This analysis revealed a cluster of brain activity in a subregion of this part of the right DLPFC (peak: *x* = 38, *y* = 48, *z* = 12 mm; *p* = 0.043; extent = 120 voxels) that revealed increased functional connectivity with the NAcc. As Fig. 4 shows, there was an altered functional coupling for high-calorie versus low-calorie food under high load in comparison to low load. More specifically, under low load the anticorrelation between NAcc and right DLPFC was stronger for high-calorie than low-calorie pictures, an effect that disappeared under high load. Analyses on the extracted PPI values using repeated-measures analyses of variance did not reveal additional significant effects. Exploratory whole-brain analyses for this contrast and its inversion did not reveal modulation in other brain regions.

#### Brain-behavior correlations

We also investigated whether the effect of load on altered neural processing of high-calorie versus low-calorie pictures correlated with the behavioral effects in the food-categorization task. We analyzed Spearman’s rho correlations between the extracted brain activity values reported above and the behavioral interaction effects in reaction time and accuracy. No significant brain-behavior correlations were observed (*p*s > .193).

## Discussion

The present fMRI study examined the influence of cognitive load on neural reward responses to food stimuli. Participants categorized pictures of high-calorie and low-calorie foods versus objects while their working memory was taxed using a digit-span task that varied in cognitive load (memorizing seven digits versus one digit) which was hypothesized to attenuate the neural reward response in the NAcc to high-calorie compared to low-calorie stimuli via interactions with DLPFC.

In line with predictions, high compared to low cognitive load engaged dorsolateral prefrontal cortex (DLPFC), suggesting that this brain region supports the active maintenance of the digits in working memory (Erk et al., 2007; Owen, McMillan, Laird, & Bullmore, 2005). Importantly, cognitive load also modulated responses to the subsequently presented high-calorie versus low-calorie food pictures in the NAcc, a region commonly reported to be involved in the processing of the hedonic relevance of perceptual cues (Berridge, 2009; Volkow et al., 2011), and more specifically to show greater reactivity in response to pictures of high-calorie versus low-calorie foods (e.g., Demos et al., 2012; Passamonti et al., 2009). Whereas the NAcc responded more strongly to high-calorie food pictures compared with low-calorie food pictures under low load, this was not the case under high load where the pattern even reversed. Notably, this modulation was observed in the first block of the categorization task, but not in the second block. Although cognitive load varied from trial to trial, the same pictures were presented twice, once in block one and once in block two. This repeated exposure may have resulted in habituation or learning effects, an interpretation that is further supported by the finding that participants displayed overall faster categorization responses in the second block compared to the first block. Another possibility is that over time, participants became less efficient in prioritizing the digit-span task over the food-categorization task. This possibility is supported by the observation that in the second block, participants were faster but performed worse on the high-load digit-span task (i.e. displayed a speed–accuracy trade-off; Schouten & Bekker, 1967), especially when it was interspersed with the more salient high-calorie food trials (and nonfood trials; see Supplementary Materials).

Functional connectivity analyses revealed altered neural coupling between the NAcc and part of the same right DLPFC region that was also engaged during the digit-span task. Interestingly, these regions were found to be anticorrelated, possibly reflecting mutual inhibition due to competition between the different representations in these two areas. This negative functional coupling was stronger for high-calorie than for low-calorie pictures under low load, an effect that disappeared under high load. Taken together with the overall modulation of nucleus accumbens activity, this finding suggests that increasing working memory load not only dampens the reward response to high-calorie food pictures but also reduces neural competition between hedonic representations of food in the NAcc and digit-span representations in the DLPFC.

Our findings contribute to the growing literature on the dynamic modulation of affective processing by higher order cognitive brain mechanisms (Erk et al., 2007; Van Dillen et al., 2009; see, for an overview, Okon-Singer, Hendler, Pessoa, & Shackman, 2015; Okon-Singer, Lichtenstein-Vidne, & Cohen, 2013), and, more broadly, to the notion that emotion is strongly embedded in cognition (Kavanagh et al., 2005; Pessoa, 2013). Our results also revealed a possible neural mechanism that might underlie recent behavioral findings showing that blocking people’s mental resources while they are exposed to attractive food cues can reduce cravings in response to these cues (Kemps et al., 2008; Skorka-Brown et al., 2014), as well as subsequent craving-induced consumption choices (Van Dillen & Andrade, 2016; Van Dillen et al., 2013).

It is important to note that the modulation of NAcc activity by cognitive load was observed in the absence of a clear behavioral effect in the food categorization task. Whereas performance on the digit-span task was influenced by the number of digits, with more errors and longer response latencies to seven digits compared to one digit, accuracy on the food categorization task was generally high and was mostly unaffected by additional load, as were the reaction times. This suggests that participants were well able to perform the food-categorization task, even under more demanding conditions. Participants moreover categorized the high-calorie food pictures more quickly than the low-calorie food pictures, but contrary to previous findings (Van Dillen et al., 2013), regardless of concurrent cognitive load. One explanation for the absence of any effects of cognitive load on the food-categorization task in the present study might be that we varied load within participants, rather than between participants. This might have resulted in learning effects and/or transfer between the various trial types. In addition, response latencies commonly reflect the outcome of varying mental processes, such that latency differences may indicate different processes under different circumstances (Bartholow, 2010; Krajbich, Bartling, Hare, & Fehr, 2015).

After the scanning session, participants did rate the high-calorie food pictures as more attractive than the low-calorie food pictures. Greater self-reported sensitivity to food rewards, as measures by the Power of Food scale, moreover related to higher attractiveness ratings of the high-calorie foods but not the low-calorie foods, suggesting that participants did differentiate between the hedonic qualities of the various food pictures they had been exposed to.

An important question to be addressed in future research is to what extent the current findings reflect actual down-regulation, or the absence of up-regulation of NAcc by the DLPFC under high versus low cognitive load. Given that the we observed reduced NAcc responses to high-calorie versus low-calorie pictures under high load, it is possible that increasing cognitive load actually induces a control state that actively inhibits emotional processing. This explanation would fit with earlier studies suggesting that the DLPFC plays an important role in self-control processes (Hare, Camerer, & Rangel, 2009; Wagner, Altman, Boswell, Kelley, & Heatherton, 2013) and recent work that has shown an association between cognitive control states and reduced processing of rewards (Veling, Aarts, & Stroebe, 2013).

On the other hand, studies have also pointed to the involvement of DLPFC in the further elaboration of motivationally relevant cues (Erk et al., 2007; Van Dillen et al., 2009; see, for a review, Goldstein & Volkow, 2011). Combining transcranial magnetic stimulation and functional magnetic resonance imaging, Hayashi, Ko, Strafella, & Dagher (2013) have demonstrated that the DLPFC modulates craving in response to changes in intertemporal availability. Subjective craving was greater when cigarettes were immediately available, but this effect was eliminated by transiently disrupting the DLPFC with transcranial magnetic stimulation. Stimulation of the DLPFC also reduced craving-related signals in the anterior cingulate and ventral striatum, areas implicated in transforming value signals into action (Walton, Devlin, & Rushworth, 2004). These findings indicate that the DLPFC is involved in the construction of value signals as much as it might be involved in the down-regulation of these signals.

Future research could also examine to what extent the current findings translate to situations where cognitive load is induced *following* rather than *before* presentation of rewarding food cues. Whereas people may engage themselves in distracting activities, in order to “turn a blind eye” to temptation (Van Dillen et al., 2013), often the encounter of such cues cannot be anticipated. The question thus rises, to what extent the current pro-active DLPFC-striatal control mechanism can also be engaged *in reaction* to exposure to reward cues. Recent neuroimaging and behavioral findings suggest that this may well be the case: Van Dillen et al. (2009), for example, demonstrated that load-induced DLPFC activity coincided with the down-regulation of responses in the amygdala and insula to pictures of negative scenes, even when cognitive load was induced *following* rather than during presentation of the affective stimulus. Harris, Hare, and Rangel (2013), moreover, demonstrated that the DLPFC is critically involved in both early attentional filtering and later value modulation of responses to appetitive food items. Although more research is needed in this area, a growing number of behavioral studies similarly point to the possibility that cognitively demanding tasks may be used to down-regulate affective influences on subjective experience (Kron, Schul, Cohen, & Hassin, 2010), memory (Crowell & Schmeichel, 2016; Holmes, James, Coode-Bate, & Deeprose, 2009), and judgment and decision-making (Gummerum, Van Dillen, Van Dijk, & López-Pérez, 2016; Van Dillen, Van der Wal, & Van den Bos, 2012).

One limitation of the current study is that our behavioral and neural measures could not differentiate between food wanting and hedonic liking. Throughout the current report, we have simply referred to the term *hedonic* as pertaining to reward, in this case from palatable food cues. Whereas the two typically co-occur, wanting and liking components have been dissociated in animal research (Berridge, 2009), where it has been shown that animals can be motivated (i.e., invest effort) to consume rewarding substances such as sucrose solutions, without obtaining pleasure from its actual consumption. Even though similar effects have been suggested in humans, making such a distinction in humans has proven to be difficult, both at the neural and behavioral level (Pool, Sennwald, Delplanque, Brosch, & Sander, 2016). For instance, even symbolic representations of food, such as images, elicit neural simulations of actual consumption (Simmons, Martin, & Barsalou, 2005) and self-reported liking of food stimuli often reflect both past consumption experiences, cravings, as well as anticipated pleasure (Pool et al., 2016). Thus, we believe that the current experiment likely probed a delicate mixture of wanting and liking responses to palatable foods. Disentangling how cognitive load would (differentially) affect wanting and liking components at the neuropsychological level would be an empirical challenge, but one that could lead to interesting new insights.

To conclude, the current study demonstrated that cognitive load modulates hedonic responses in the brain. Connectivity analyses moreover showed that cognitive load altered the neural coupling between the NAcc and a part of the DLPFC that was increased by cognitive load. One important question for future research is to show how the modulation by cognitive load of food cue reactivity in the NAcc relates to actual food choices, to gain a better understanding of the nature of hedonic consumption and how it can be regulated.

## Electronic supplementary material

ESM 1 (DOCX 13373 kb)
