# Supplementary material for: Tuning down the hedonic brain: Cognitive load reduces neural responses to high-calorie food pictures in the nucleus accumbens
Source: Cogn Affect Behav Neurosci. 2018 Mar 14;18(3):447–59. doi: 10.3758/s13415-018-0579-3 (PMC5962628; doi:10.3758/s13415-018-0579-3)
Supplement: Supplementary file 1 — (DOCX 13373 kb) [file 13415_2018_579_MOESM1_ESM.docx]

Supplementary materials

**Behavioral analyses including object trials.**

**Digit span task.**

Table S1 depicts all means and standard errors for reaction times and accuracy scores on the digit span task and the food categorization task as a function of cognitive load (high, low), picture type (high-calorie versus low-calorie foods, non-food objects), and block (first, second). The analyses scripts and data can be found on the OSF link: <https://osf.io/e9pjm>

The GEE analysis of the digit span accuracy scores revealed significant main effects of load, Wald (1) = 72.33, *p* < .001, type, Wald (1) = 30.41, *p* < .001, and block, Wald (1) = 6.97, *p* = .008. Participants on average successfully retrieved 93% of the one-digit numbers (*SE* = 1.9%) and 65% of the seven-digit numbers (*SE* = 2%), confirming the effectiveness of our cognitive load manipulation. Participants moreover performed worse on the less frequent trials containing non-food pictures (*M* = 75%, *SE* = 2.5%) than the trials containing pictures of either high-calorie (*M* = 84%, *SE* = 2.4%) or low calorie foods (*M* = 82%, *SE* = 2.3%). Participants also made more accurate responses in the first block (*M* = 89%, *SE* = 1.9%) compared to the second block (*M* = 74%, *SE* = 5.6%) indicating that overall digit span performance deteriorated over time. The analyses additionally revealed interaction effects of load and type, Wald (2) = 20.84, *p* < .001, load and block, Wald (1) = 7.692, *p* = .005, and type and block, Wald (2) = 16.45, *p* < .001, which were further qualified by a three-way interaction between load, type and block, Wald (2) = 10.77, *p* < .001. In the first block, participants were less accurate during non-food trials compared to both high- and low-calorie food trials under low cognitive load (*p*s <.001) but not high cognitive load (*p*s >.117), suggesting that performance on the digit span task was prioritized over picture processing when working memory load was high compared to low. In the second block, however, participants were less accurate during non-food trials compared to both high- and low-calorie food trials under low load, and during both high-calorie food trials (*d* = -8%, *SE* = 2.2%, *p* < .001, *CI*: [-13, -4]) and non-food trials (*d* = -4%, *SE* = 1.6%, *p* = .018, *CI*: [-7, -1) compared to low-calorie food trials under high load, suggesting that picture content interfered more with digit span performance later on during the experiment for both the low load and high load trials.

Mixed model analyses of the reaction times on the digit span task revealed main effects of load, *F* (1, 91.99) = 730.82, *p* < .001, and block, *F* (1, 91.99) = 13.51, *p* < .001. Participants were faster to respond to the one digit (*M* = 748 ms, *SE* = 21 ms) than to the seven digit series (*M* = 1165 ms, *SE* = 20 ms) , and became faster from the first block (*M* = 985 ms, *SE* = 21 ms) to the second block (*M* = 928 ms, *SE* = 21 ms). The main effects of load and block were further qualified by an interaction effect; *F* (1, 4205) = 3.86, *p* = .049, such that the speed-up from block one to block two was much greater for low load trials, *F* (1, 172.37) = 17.31, *p* < .001, *CI*: [39, 110], than for high load trials, *F* (1, 172.37) = 4.49, *p* = .036, *CI*: [3, 74].

**Food categorization task.** The GEE analysis of participants’ accuracy scores only revealed a significant interaction of load and block, Wald (1) = 6.33, *p* = .009. Although overall differences were small, in the first block performance was somewhat better under low load (*M* = 97%, *SE* = 1.7%) than under high load (*M* = 96%, *SE* = 2.2%; Wald (1) = 4.93, *p* = .026, *CI*: [-3, 0]), whereas performance during the second block did not vary significantly across low (*M* = 96%, *SE* = 2.2%) versus high cognitive load (*M* = 97%, *SE* = 1.9%; Wald (1) = 2.69, *p* = .104, *CI*: [-1, 0]). Performance moreover deteriorated significantly from the first block to the second block for low load trials, Wald (1) = 7.89, *p* = .005, *CI*: [-2, 0], whereas this was not the case for high load trials, Wald (1) = 3.49, *p* = .062, *CI*: [-2, 0].

A linear mixed model analysis of participants’ reaction times on the food categorization task revealed significant main effects of stimulus type, *F*(2, 122.59) = 6.95, *p* = .001, and block, *F*(1, 94.27) = 104.89, *p* < .001. Participants were faster to categorize high-calorie food pictures (*M* = 686 ms, *SE* = 22 ms) than low-calorie food pictures (*M* = 720 ms, *SE* = 21 ms, *p* <.001, *CI*: [-51, -16]) and objects (*M* = 717 ms, *SE* = 21 ms, *p* = .004, *CI*: [-51, -10]), and made faster categorizations in the second block (*M* = 655 ms, *SE* = 20 ms) compared to the first block (*M* = 751 ms, *SE* = 21 ms, *CI*: [-101, -69]). In addition there were interaction effects of type and load, *F*(2, 3998.79) = 10.68, *p* < .001, and type and block, *F*(2, 3974.97) = 5.95, *p* = .003. When cognitive load was low, participants were faster to categorize high-calorie foods compared to low-calorie foods; *d* = , *SE* = , *p* =.017, *CI*: [-46, -5], but not compared to objects; *p* =.537, *CI*: [-30, 16]. When cognitive load was high, participants were faster to categorize high-calorie foods compared to both low-calorie foods; *d* = , *SE* = , *p* <.001, *CI*: [-63, -22], as well as objects; *d* = , *SE* = , *p* <.001, *CI*: [-78, -31]. Categorization speed of low-calorie foods did not differ significantly from that of objects under both low cognitive load, *p* =.090, *CI*: [-2, 38], and high cognitive load, *p* =.273, *CI*: [-9, 32].

When standardized power of food scores or BMI were included as covariates in the above analyses, this yielded no additional significant effects and did not alter the above-reported pattern of findings.

**Effect of picture type independent of load on neural activity**

We also assessed the effects of picture type independent of cognitive load at the whole-brain level. As Figure S1 shows, food > non-food pictures activated ventromedial prefrontal cortex, visual cortex, motor cortex and cerebellum (upper panel). High-calorie > non-food pictures (middle panel) involved visual and motor cortex. High-calorie > low-calorie pictures activated visual cortex (lower panel).

**Extracted NAcc activity as a function of picture type, load and block.**

For reasons of completeness, we also analyzed the extracted brain activity from the NAcc cluster observed in our main analysis as a function of all levels of food type, load, and block. As Figure S2 shows, this analysis showed that the non-food pictures were associated with lower NAcc activity than the low-calorie pictures under high load in the first block. However, in the same block non-food in comparison to low-calorie pictures were not associated with more NAcc activity under low load. One possible interpretation of this finding is that NAcc activity to non-food pictures reflects differences in salience processing of the stimulus, as non-food pictures were presented less frequently than food pictures. Other studies indeed have shown that the NAcc is not only responsive to appetitive information, but that it may also signal novelty and contextual deviance (Zaehle et al. 2013).

**Table S1. Performance on the digit span task and food categorization task.**

Means and standard errors (between brackets) for reaction times (RTs) in milliseconds and accuracy scores in percentages, and as a function of cognitive load (high, low), target picture (high-calorie vs low-calorie food, non-food objects) and block (first, second).

|  | | | **Digit span** | | **Food categorization** | |
| --- | --- | --- | --- | --- | --- | --- |
|  |  |  | **RTs** | **Accuracy** | **RTs** | **Accuracy** |
| High load | High-cal. | Block 1 | 1186 (27) | 69 (2) | 717 (22) | 95 (3) |
|  |  | Block 2 | 1155 (28) | 59 (3) | 638 (21) | 98 (1) |
|  | Low-cal. | Block 1 | 1185 (28) | 65 (2) | 763 (21) | 94 (4) |
|  |  | Block 2 | 1128 (27) | 67 (3) | 671 (20) | 97 (2) |
|  | Non-food | Block 1 | 1181 (27) | 66 (2) | 764 (23) | 90 (2) |
|  |  | Block 2 | 1155 (26) | 63 (3) | 694 (22) | 93 (3) |
| Low load | High-cal. | Block 1 | 770 (27) | 98 (1) | 745 (21) | 95 (3) |
|  |  | Block 2 | 695 (28) | 82 (7) | 656 (22) | 98 (1) |
|  | Low-cal. | Block 1 | 790 (28) | 96 (1) | 775 (21) | 94 (3) |
|  |  | Block 2 | 687 (27) | 83 (6) | 674 (21) | 98 (1) |
|  | Non-food | Block 1 | 799 (28) | 86 (1) | 736 (23) | 97 (1) |
|  |  | Block 2 | 752 (27) | 79 (7) | 678 (23) | 90 (2) |


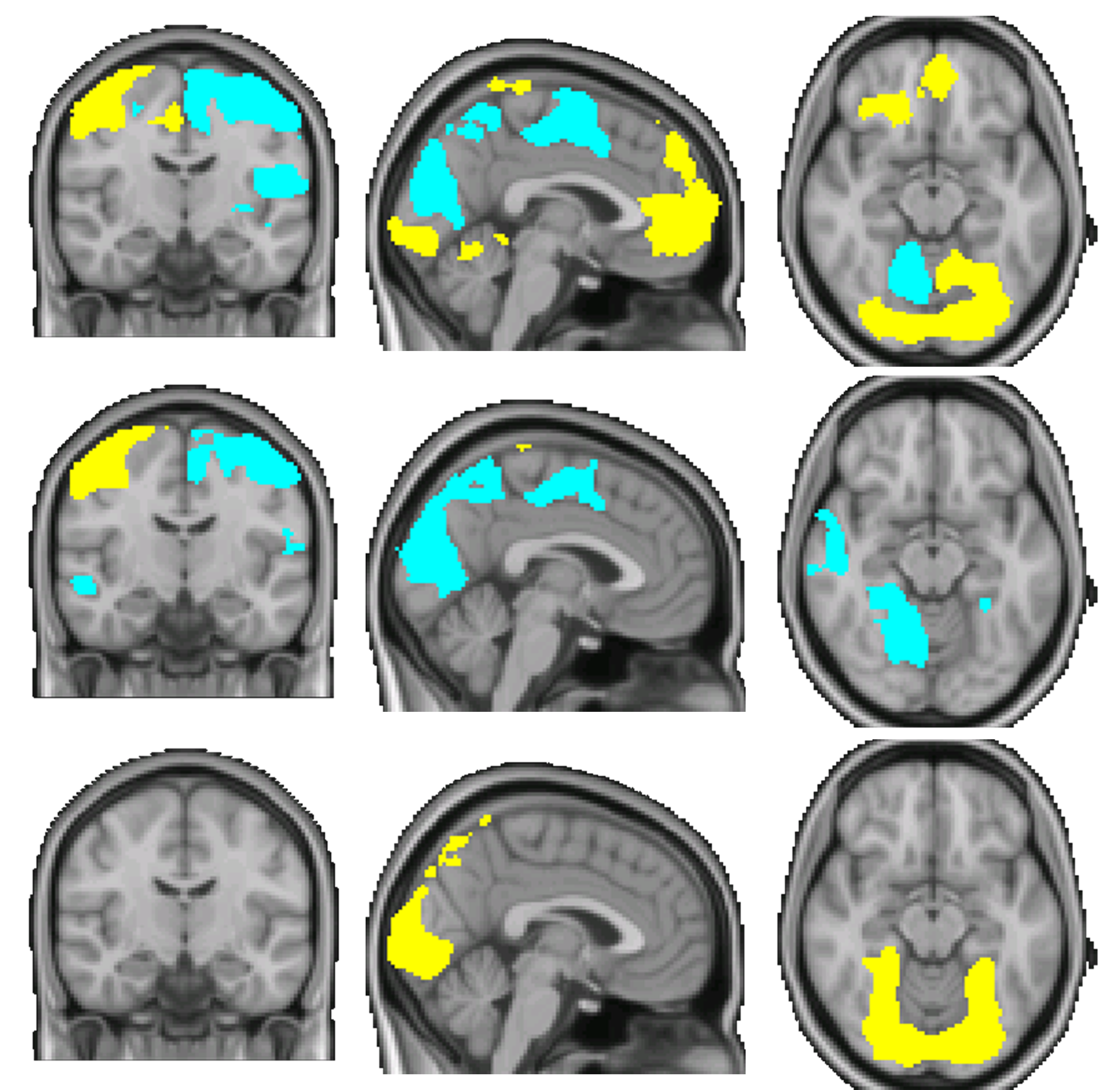


**Figure S1.** Whole-brain analyses for the contrasts food > non-food (top panel), high-calorie > non-food (middle panel) and high-calorie > low-calorie (lower panel). Slices are shown at x=-2, y=-12, z=-14 mm. Activation is depicted in yellow, deactivation in blue. All figures show cluster-corrected results at the whole-brain level, p < .05.


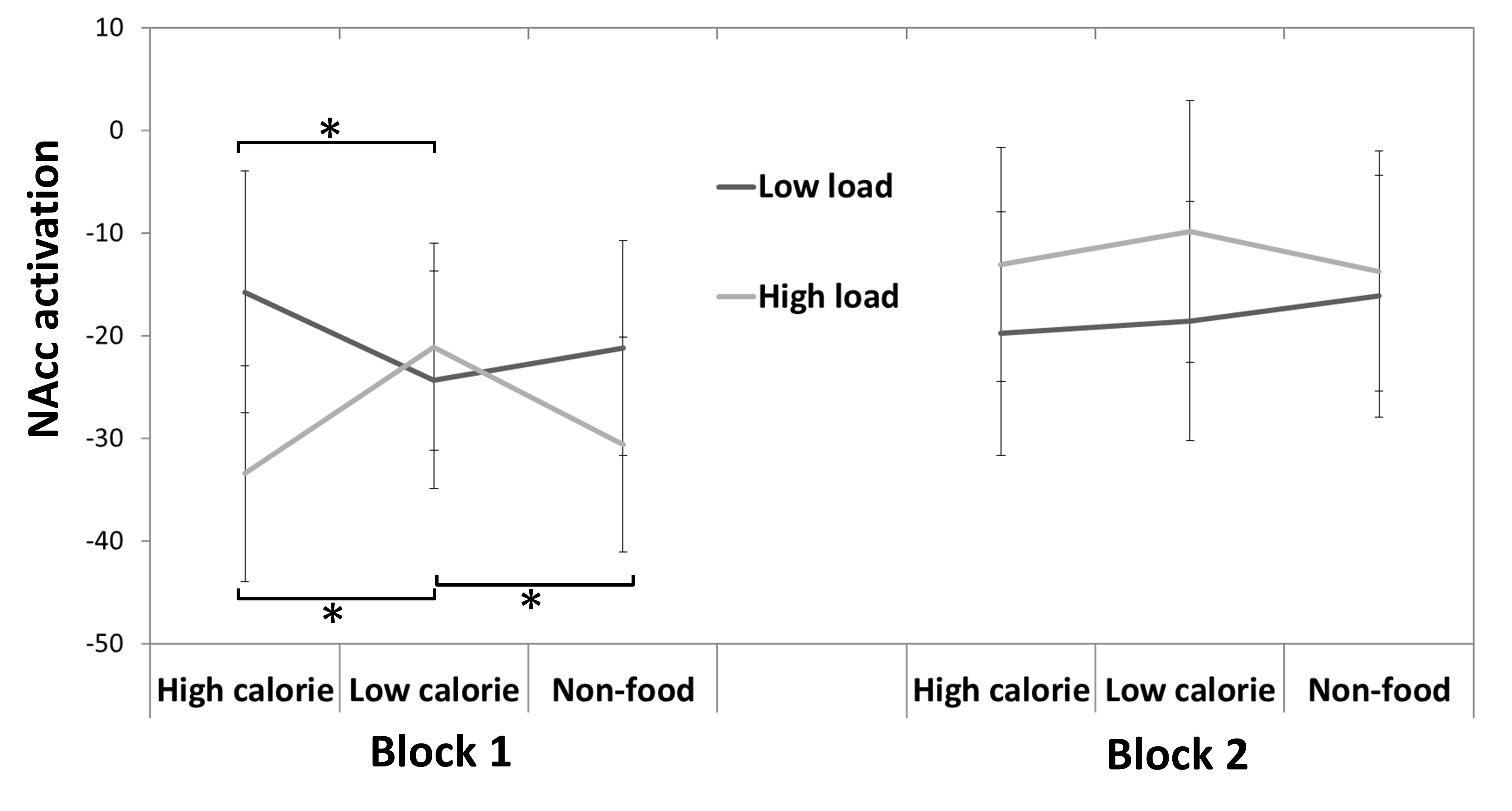


**Figure S2.** Brain activity for the cluster shown in Figure 3 in the main text as a function of all levels of stimulus type, load, and block. Asterisks in the line graph indicate significant differences within the two load conditions, p < .05. Error bars show standard errors of the means.

**Supplementary references**

Zaehle, T., Bauch, E. M., Hinrichs, H., Schmitt, F. C., Voges, J., Heinze, H. J., & Bunzeck, N. (2013). Nucleus accumbens activity dissociates different forms of salience: evidence from human intracranial recordings. *Journal of Neuroscience*, *33*, 8764-8771.
